# Manufacturability Assessment of Design Decisions for Reducing Material Diversity in Single-Piece and Small-Batch Production

**DOI:** 10.3390/ma19020399

**Published:** 2026-01-19

**Authors:** Dorota Więcek, Dariusz Więcek, Ivan Kuric

**Affiliations:** 1 Department of Production Engineering, Faculty of Mechanical Engineering and Computer Science, University of Bielsko-Biala, 43-309 Bielsko-Biała, Poland; wiecekd@ubb.edu.pl; 2 Department of Automation and Production Systems, Faculty of Mechanical Engineering, University of Zilina, 010 26 Zilina, Slovakia; ivan.kuric@fstroj.uniza.sk

**Keywords:** production management, design for manufacturability, reduction in material diversity, alternative materials, single-piece production, small-batch production, activity-based costing (ABC), cost optimisation, material management

## Abstract

**Highlights:**

**What are the main findings?**

- A manufacturability assessment method focused on reducing material diversity was developed.
- Alternative material use was analysed considering machining and material management costs.
- An integrated model combining design, process planning and ABC costing was proposed.

**What are the implications of the main findings?**

- Material assortment can be reduced without violating technological constraints.
- Local increases in machining cost may lead to overall system-level cost savings.
- The method supports intelligent decision-making in single-piece and small-batch production.

**Abstract:**

The article presents a method that supports the evaluation of design manufacturability in the area of input material selection, enabling the reduction in material diversity under single-piece and small-batch production conditions. The proposed approach combines the analysis of alternative materials with activity-based costing (ABC) and data concerning actual and planned material requirements. The method enables the assessment of the impact of semi-finished product substitution on material costs, processing costs, production organisation, and material-management costs before order execution is launched. In the conducted case study, it was demonstrated that effective management of material diversity can significantly reduce the range of materials and decrease total manufacturing costs. For the analysed period, the number of material items was reduced from 32 to 19 (a 41% reduction), resulting in cost savings of approximately 11,000 PLN. In addition to total cost, the approach supports the assessment of operational benefits associated with reduced material diversity, such as a lower number of material items, fewer suppliers, reduced inbound inspection and receipt operations, and decreased inventory levels and capital tied up in stock. Material substitution may decrease or increase direct material costs and may increase machining time when larger dimensions are used; therefore, the method jointly evaluates cost and lead-time impacts prior to order release. The results confirm that integrating design, technological, and logistics data is an effective approach to rationalising material management in machinery manufacturing enterprises.

## 1. Introduction

Managing a modern manufacturing enterprise requires the simultaneous control of multiple interacting streams: material, information, and financial. The complexity of these interdependencies becomes particularly evident in machinery industry companies, where production is carried out under single-piece or small-batch conditions. High order variability and a wide assortment of materials necessitate the application of advanced methods for resource planning and optimisation [1,2,3,4,5,6,7].

In the classical approach to procurement, priority was given to the price-to-quality ratio of purchased materials. Currently, decisions related to material selection must be made in an interdisciplinary manner, involving designers, technologists, production planners, purchasing departments, and management accounting. Designers increasingly seek to use semi-finished products that are as close as possible to the final part geometry, which reduces machining effort but simultaneously increases the purchase cost of materials [8,9,10,11]. Conversely, purchasing departments aim to limit excessive quality requirements and material variety in order to rationalise material costs. In practice, maintaining a balance between these objectives is not always feasible [12,13,14,15].

Under single-piece and small-batch production conditions, enterprises often maintain a very wide assortment of material grades, forms, and dimensions. This leads to high costs associated with ordering, transportation, delivery control, storage, and capital tied up in inventory. These difficulties are further intensified by the fact that many materials are used only sporadically, while suppliers frequently offer them exclusively in standard dimensions [5].

In such environments, excessive material variety additionally amplifies demand volatility at the item level, as numerous rarely used material items must be planned and replenished irregularly, increasing randomness in procurement and production planning. A rational response is to reduce the number of assortment items—by grade, form, condition, and dimensions—through the use of alternative materials that do not adversely affect product functionality. Consolidating material demand by reducing the number of material items and using admissible substitutes stabilises procurement processes and simplifies production scheduling. However, material substitution may require additional machining operations or longer processing times. Therefore, any reduction in material diversity must be supported by an analytical assessment ensuring that accepted substitution variants satisfy production lead-time and due-date constraints, thereby preventing delays in order completion. At the same time, decisions regarding the use of substitutes should be preceded by an analysis of their impact on production costs and on the structure of manufacturing processes. Consequently, it becomes necessary to optimally limit the assortment of materials ordered in a given period in order to minimise total production costs. Modern enterprises must consider a wide range of interrelated factors, including technological operation costs, the availability of substitutes, storage costs, the number of suppliers, production process flexibility, and the current production schedule [5,8,14,16].

The aim of this article is to evaluate the manufacturability of design decisions with respect to the potential for reducing material diversity and to present a method for optimising material requirements in enterprises engaged in single-piece and small-batch production. The developed method is based on Activity-Based Costing (ABC) analysis and cost drivers, as well as on data concerning actual and planned material requirements. This enables the simulation of the effects of decisions regarding changes in semi-finished product and the selection of alternative materials before order execution is launched.

In this study, the evaluation of manufacturability of design decisions is understood as a Design-for-Manufacturability (DFM) assessment, that is, an evaluation of how design decisions concerning the selection of input semi-finished products and admissible substitutes affect manufacturing cost, processing time, and material-management complexity prior to order release.

Classical Activity-Based Costing (ABC) applications focus primarily on improving the accuracy of cost allocation and post hoc cost analysis. The method proposed in this study integrates Activity-Based Costing with a preliminary material requirements plan and a database of alternative materials. This integration enables the evaluation of design manufacturability from the perspective of material diversity reduction at an early design and production planning stage, before material purchasing decisions are made. The method explicitly accounts for the impact of material substitution on direct material costs, processing activity costs, logistics, inventory-related costs, and production lead time.

The novelty of the approach lies in the dynamic linkage between design decisions related to material selection and the current structure of the company’s material requirements. This feature is particularly relevant in single-piece and small-batch production environments characterised by high demand variability. The proposed method allows for the generation and evaluation of alternative material requirement variants under real production constraints, supporting informed decision-making aimed at reducing material assortment while maintaining acceptable cost and time performance.

The proposed method is based on a structured set of cost drivers and on the appropriate estimation of their values, enabling cost estimation for a given planning period that closely reflects actual manufacturing costs. The calculations are performed using current activity cost rates, without relying on historical activity costs from previous periods.

## 2. Materials and Methods

The research was conducted in a machinery manufacturing enterprise operating under single-piece and small-batch production conditions. The company is characterised by high variability in material requirements and by the use of a broad assortment of steel bars with different grades and diameters. For example, in a given year, demand was recorded for 23 steel grades; for one of them (40HM), demand covered 38 different diameters. This illustrates the scale of material diversity that must be managed. High demand variability results in frequent replenishment of inventory in small batches, which generates substantial logistics and storage costs.

In the analysed enterprise, material costs account for approximately 60% of total production costs. Prior to this study, product costs were calculated using a traditional absorption costing system based on a limited number of generalised allocation keys. This approach led to significant distortions in product cost estimates, as it failed to reflect the actual structure of activities and cost drivers, particularly under single-piece and small-batch production conditions.

Internal company analyses showed that, for a sample of ten production orders, the average deviation between estimated and actual costs using traditional costing was approximately 31%, with systematic underestimation in all cases. The largest deviations concerned material costs (79.7%), followed by labour and workstation-related costs (13.8%) and indirect costs (10.3%). These results provided practical justification for adopting Activity-Based Costing in the proposed method. Traditional costing approaches proved inadequate for reliable cost estimation in the analysed production environment. In contrast, the ABC-based approach enables more accurate and transparent cost assignment by explicitly linking costs to activities and their underlying cost drivers. This is particularly important when evaluating alternative material-selection scenarios at the design stage.

**Figure 1:**
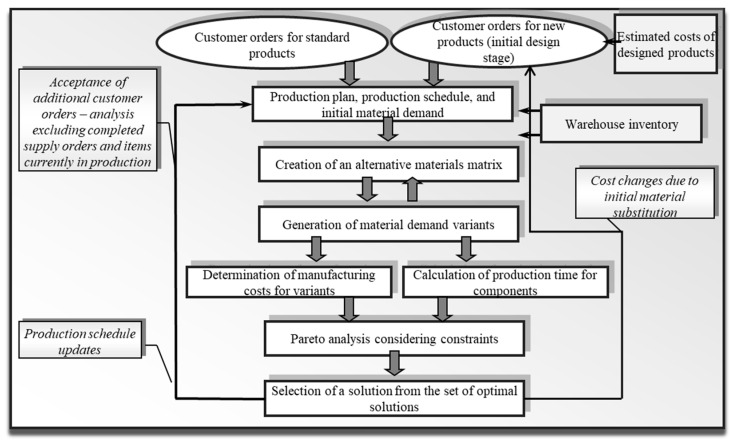
Model for determining material requirements.

For the analysed enterprise, a method was developed that—at the stage of estimating the costs of the designed product and making decisions about its production—enables further optimisation of both the design and the production process according to cost criteria, taking into account the current material requirements plan [17]. Conducting simulations of alternative decisions regarding the selection of input materials makes it possible to estimate the manufacturing cost level even before decisions are made and resources are purchased (Figure 1).

Solving the problem required the following elements [8,17]:

- The use of tools enabling the measurement and estimation of costs for newly designed products;
- The identification of activities and the determination of their costs and cost drivers;
- The use of a preliminary material requirements plan derived from accepted production orders for a given planning period, taking current inventory levels into account;
- The application of databases defining the possibility of replacing selected semi-finished products with alternative materials;
- The analysis of the impact of material substitution on manufacturing costs, production lead time, and the structure of manufacturing processes;
- The determination of the total lead time of production orders when alternative materials are applied.

The starting point was the appropriate application of Activity-Based Costing, with the number of allocation bases corresponding to the number of identified activity types (Table 1). Activity costs should be properly allocated at every level of the product structure (assemblies, subassemblies, components, and elementary objects). This approach enables the differentiation of costs between alternative design concepts [17,18,19].

Based on data from previous periods available in the financial and accounting systems, as well as information on the occurrence and intensity of individual activities in the production system, direct activity costs were determined (an excerpt is presented in Table 1). After establishing direct activity costs and the associated activity volume measures, current cost rates for individual activities were calculated [20,21,22].

**Table 1:** Excerpt from direct activity costs [23].

Activity	Direct Activity Cost PLN	Indirect Activity Cost PLN	Total Activity Cost PLN
Order acceptance	29,200	71,486	100,686
Preparation of documentation—standard product	6832	2120	8952
…			
Activities at the robotic cutting station	11,846	9790	21,632
Activities at the robotic milling centre	95,936	54,214	150,150
Activities at the robotic turning centre	73,852	55,884	129,726
Activities at the autonomous machining station	76,184	56,498	132,692
Activities at the robotic 3D printing station	55,020	25,600	80,620
…			
Component storage	6902	2618	9520
Component inspection	60,082	12,432	72,514
…	…	…	…

In the course of the research, there was developed a model (Figure 1) that integrates the following elements:

1. Analysis of the design with regard to the possibility of using alternative materials

A matrix of alternative materials was created, taking into account permissible substitutes within defined groups of material properties and dimensions.

2. Analysis of material standards (MS)

For each variant, the material standard was determined based on the following relationship:

(1) $${MS}_{w}=f(alternative\;diameter,length,density),$$

When an alternative material was applied, it was necessary to determine the extent to which this substitution affects the components of direct material costs, namely the updated material standard ${MS}_{wy}$ and the unit price of the alternative material. For example, the requirement for the new material ${MS}_{wy}$, depending on the diameter (outer dimension) of the original material $D_{y}$ and the substituting material $D_{w}$, as well as the requirement for the original material ${MS}_{y}$, can be determined as follows:

(2) $${MS}_{wy}={\left(\frac{D_{w}}{D_{y}}\right)}^{2}{\cdot MS}_{y},$$

where $D_{y}$ and $D_{w}$ denote the outer dimensions of the original and substituting materials, respectively. Equation (2) updates the material standard after substitution by reflecting the geometric change in a round bar.

3. Assessment of the impact of changing the semi-finished product on manufacturing costs and time:
  - Direct material cost

The updated ${MS}_{w}$ is multiplied by the substitute unit price $p_{w}$, which directly quantifies the material cost impact of substitution. The direct material cost $C_{DM}$ per unit was determined using:

(3) $$C_{DM}=\sum_{w=1}^{W}{MS}_{w}\cdot p_{w},$$

where

${MS}_{w}$—material standard for material w,

$p_{w}$—unit price of material w.

  - Time and cost of technological operations

The company uses automated and robotic modules that can replace or support workers in performing operations. Therefore, direct labour costs are included as processing activity costs, which depend on the operation of machining and assembly modules and their supervision. Technological operation costs were determined based on machine time standards before and after material substitution (${ST}_{My}$, ${ST}_{Mw}$). For each operation, the processing activity cost $C_{AP}$ was calculated as:

(4) $$C_{AP}=\sum_{w=1}^{W}{ST}_{Mw}\cdot C_{M},$$

where:

${ST}_{Mw}$—machine time standard after substitution of semi-finished product w,

$C_{M}$—machine cost rate.

The machine time standard ${ST}_{M}$, associated with performing an operation, is expressed as:

(5) $${ST}_{M}\;=\;Tu+\frac{Tsf}{b},$$

where:

Tsf—setup and finishing time of the operation or mini-operation sequence s,

*b*—batch size,

Tu—unit operation time at production workstation s.

The unit time standard ${Tu}_{Mw}$ after material substitution was determined as:

(6) $${Tu}_{Mw}\;=\;{Tu}_{My}\cdot \frac{\frac{1}{q^{\lambda -\mu }}+c_{aux}}{1+c_{aux}},$$

where:

${Tu}_{My}$—unit time before material substitution,

$c_{aux}=0.08$—auxiliary time coefficient,

*λ*, *μ*—workpiece material groups,

*q* = 1.26.

Post-substitution time standards are calculated as follows. The baseline unit time ${Tu}_{My}$ and setup/finishing time Tsf were taken from the company’s routing standards. The substitute unit time ${Tu}_{Mw}$ was recalculated using Equation (6) based on material-group correction factors. Subsequently, the operation time standard ${ST}_{M}$ was obtained from Equation (5), where ${Tu}_{Mw}=Tu.$ These time standards were then used in Equation (4) to compute processing activity costs.

  - Process organisation

Production tasks were assigned to the resources of the manufacturing system in accordance with its actual technological capabilities [24].

  - Inventory management

The reduction in material diversity was analysed with respect to the number of orders and deliveries, transport costs, storage and capital tie-up costs, and the number of quality control operations for incoming materials.

After selecting a feasible substitute from the alternative materials matrix, the updated material standard and direct material costs are calculated, together with changes in processing activity costs, including potential additional operations (e.g., extra cutting or turning operations when using materials of larger dimensions), as well as changes in material management costs resulting from assortment consolidation. Taken together, these components form the total cost difference used to compare variants under lead-time constraints.

4. Optimisation of material requirements

The optimisation of material requirements was carried out using a deterministic, rule-based variant-generation procedure integrating material, technological, cost, and time-related constraints.

The input data included:

- The production order schedule;
- Current inventory levels;
- A set of admissible alternative materials;
- Cost data covering materials, processing activities, ordering, transportation, and storage.

In addition, technological feasibility, quality requirements, and due dates of production orders were considered.

A variant-generation algorithm for material requirements was applied. Firstly, we determine a binary matrix $M=[m_{j,k}\;]$ describing the possibility of replacing a material with an alternative: if a material j can be replaced by alternative k, the corresponding element $m_{j,k}=1$; otherwise $m_{j,k}=0$. The preliminary materials are arranged in an order according to their dimensions (from the largest to the smallest diameter). The matrices $X^{n}=\left[x_{i,l}^{n}\right]$—describe the n generated variants for i=1,2 and l=1,…,L. Row i=1 defines the semi-finished products resulting from the preliminary material demand, whereas row i=2 defines the new semi-finished product requirements for a given variant, taking into account the available alternative materials.

The matrices were filled inductively. For each possibility at step l, two variants were generated:

- Variant 1 consists of placing a separate order for the given material item and appending it to the solution from the previous step, i.e.,

(7) $$x_{1,l+1}=x_{2,l+1}.$$

- Variant 2 consists of replacing the given material item in each variant with the preceding semi-finished product, i.e.,

(8) $$x_{1,l+1}=x_{2,l},$$

The second variant was considered only if the corresponding element $m_{w,y}$ in matrix M, associated with materials $x_{1,l+1}$ and $x_{2,l}$ was equal to 1, indicating that substitution was feasible (i.e., material $x_{1,l+1}$ can be replaced by $x_{2,l}$).

Thus, at each step l, at most two new variants were generated from each existing variant. Consequently, for L materials, the total number of possible variants did not exceed $2^{L-1}$.

As a result of this procedure, all feasible solutions corresponding to the initial material demand were generated.

In the next step, the total costs associated with each variant were calculated. The total manufacturing cost $C_{t}(v)\;$ for variant v was determined as the sum of direct material costs $C_{DM}(v)$, processing activity costs $C_{AP}(v)$ calculated using Activity-Based Costing, and material management costs $C_{AMM}(v)$. In parallel, the total execution time of production orders was computed based on updated machine time standards resulting from material substitution.

(9) $$C_{t}\left(v\right)=C_{DM}\left(v\right)+C_{AP}\left(v\right)+C_{AMM}\left(v\right),$$

The optimisation criteria for selecting material items in the requirements list were:

- Minimization of the total manufacturing costs of production orders forming the basis of the material requirements plan;
- Minimization of the total lead time of production orders.

The constraints defining the admissible solution set were:

- $T_{e}(v)\leq T_{or}$, where $T_{e}(v)$ is the total execution time of components for variant v and $T_{or}$ is the due date of the orders;
- $C_{tot}(v)\leq C_{pre}$, where $C_{tot}(v)$ is the total cost of executing orders for variant v and ${\;C}_{pre}\;$ denotes the total cost of the preliminary material requirements.

The variant-generation process was terminated after all combinations satisfying these constraints had been evaluated.

From the generated variants, there was identified the Pareto frontier (the set of non-dominated solutions) $P^{*}$, i.e., the set of variants $v^{*}$, such that for all other variants v there is either $C_{tot}(v^{*})\leq C_{tot}\left(v\right)$ or $T_{e}(v^{*})\leq T_{e}(v)$. All solutions $v^{*}\in \;P^{*}$ were considered feasible for implementation. The final variant was selected based on secondary criteria, including material availability, technological constraints, and quality requirements [25,26].

## 3. Results

### 3.1. Example of Cost Reduction

To illustrate the operation of the developed method, an example analysis of demand for steel bars—one of the main groups of semi-finished products used in the considered company—is presented. The analysis was carried out based on actual and planned production data. Using the production schedule, the preliminary demand for steel bars for the selected planning period was compiled (Table 2). The binary matrix defining the feasibility of substituting individual semi-finished products with admissible alternatives is presented in Table 3.

In the next stage, variants of material requirements were generated using alternative materials. Due to the large number of generated variants, the presented example was limited to six selected semi-finished products. Table 4 presents the corrected variants obtained using the above binary matrix. The first column lists the variant number, the second column shows the semi-finished products included in the preliminary material requirements, and the last column presents the alternative semi-finished products included in the analysed material requirement variants.

In the subsequent step, after generating all admissible variants, the total costs associated with each solution were calculated. These costs included direct material costs, processing activity costs, and material procurement and management costs.

The application of alternative materials resulted in the following effects:

- Changes in direct material costs, driven by an increased material standard but, in some cases, a lower unit price of the substitute material. Table 5 presents an excerpt from the analysis of changes in direct material costs ${\Delta C}_{DM}$, resulting from the material standard before substitution ${MS}_{y}$ and after substitution ${MS}_{w}$, unit prices of semi-finished products $p_{y}$ and $p_{w}$, and the corresponding direct material costs $C_{DMy}$ and $C_{DMw}$.
- An increase in processing-related activity costs, resulting from dimensional and technological differences between the original and substituted materials (Table 6 and Table 7).
- A reduction in ordering, transportation, and material receipt costs, resulting from a reduced number of assortment items, fewer suppliers, and the consolidation of material requirements (Table 8).
- A decrease in warehousing costs and capital tied up in inventory, due to a lower number of stock-keeping units and reduced safety stock levels (Table 9).

Table 6 presents detailed calculations for a sample semi-finished product (3H13 bar). It includes the technological operations, machine time standards before and after substitution $({ST}_{My}$_,_ ${ST}_{Mw}$), the processing activity cost rate Cmh, the processing activity costs assigned to the part before and after substitution $C_{APy}$ and $C_{APw}$ and the resulting difference in processing costs ${\Delta C}_{P}$.

Table 7 summarises the additional turning costs $C_{APad.}$ arising from the substitution of the original material with a larger-diameter alternative. After adding these additional costs to the processing cost changes ${\Delta C}_{P}$, the total processing cost $C_{P}$ was obtained.

Table 8 presents the material management costs $C_{AMM}$, including ordering, transportation, receipt, and storage activities assigned to the given item. For repeated material items, these costs were counted only once. The table also summarises the differences in direct material costs ${\Delta C}_{DM}$, processing costs $C_{P}$ and material management costs ${\Delta C}_{AMM}$, as well as the total cost difference ${\Delta C}_{T}$.

**Figure 2:**
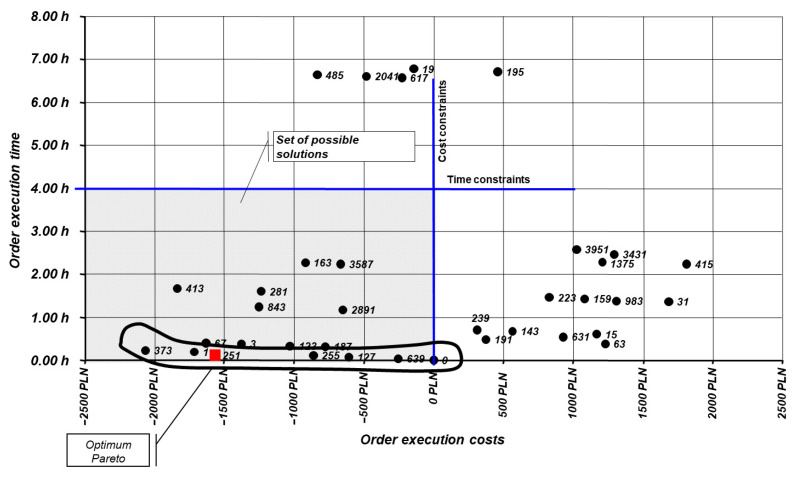
Pareto analysis.

Table 9 presents an excerpt from the reduced material requirements plan. In the first analysed period, the assortment of 13 items was reduced by four items, resulting in a total manufacturing cost reduction of approximately 1550 PLN (variant no. 251). In the subsequent step, the differences in execution times for parts manufactured from individual semi-finished products was calculated for the generated variants. A Pareto analysis was then performed (Figure 2) taking into account cost and time constraints. From the set of non-dominated solutions, the final variant was selected based on secondary criteria, namely material availability, technological constraints, and quality requirements.

### 3.2. Results of Material Assortment Reduction

In the initial production plan for the analysed period, 32 different steel bar items with varying diameters and grades were included. Analysis of the alternative materials matrix revealed numerous feasible substitution relationships between semi-finished products, enabling a substantial reduction in the material assortment.

The application of alternative materials affected:

- Increases or decreases in direct material costs ΔC_DM_;
- Increases in processing costs resulting from machining larger-diameter workpieces;
- Reduction in ordering, storage, and capital tied up in inventory.

For example, replacing a 3H13 bar with a diameter of 110 mm with a 1H18N9T bar of 140 mm diameter resulted in:

- An increase in material allowance and direct material cost of 110.49 PLN per item;
- An increase in processing costs of 168.00 PLN per item;
- A simultaneous reduction in material management costs due to a lower number of assortment items.

#### 3.2.1. Effect of Assortment Reduction

In the first analysed period, the number of material items was reduced by four, resulting in savings of approximately 1550 PLN. On a monthly basis, the reduction amounted to:

- A decrease from 32 to 19 material items (a 41% reduction);
- Approximately 11,000 PLN in total savings;
- A reduction in the number of suppliers;
- Fewer delivery receipt and inspection operations;
- Lower safety stock levels.

#### 3.2.2. Selection of the Optimal Variant

Among the hundreds of generated material requirement variants, the solution was selected that simultaneously ensured:

- The lowest total manufacturing cost;
- Compliance with production lead-time constraints;
- Rationalisation of material management.

The selected variant was subsequently used to prepare the final material requirements plan for the analysed period.

## 4. Discussion

The conducted research confirms operating under single-piece and small-batch production conditions possess significant cost-reduction potential through limiting material diversity. Reducing the number of material grades, forms, and dimensions has a strong impact on several key areas [27], including:

- Improved supply logistics;
- Lower storage costs;
- Changes in processing costs;
- Simplified production planning;
- Improved efficiency of resource utilisation.

However, the use of alternative materials requires careful consideration. Replacing a semi-finished product may lead to an unfavourable increase in machining costs, extended order lead times, or the need to introduce additional technological operations. Therefore, such decisions should be supported by advanced analytical tools that enable the consideration of complex interdependencies between product design, manufacturing technology, and production organisation [13,17,28].

The optimisation process should be dynamic and adjusted to current production conditions. From the perspective of technological maturity, effective implementation of the proposed method requires at least a basic level of digital integration, including ERP systems for material and inventory management, CAPP systems for process planning, and reliable activity-based cost data. Although full production automation is not required, the availability of accurate time standards and process data significantly improves the precision of cost and lead-time estimation. These tools are essential for analysing processes holistically rather than in isolation—for example, focusing solely on procurement—which does not yield the desired results [2,23,29,30,31].

Modern enterprises should also strive for close integration of design, manufacturing engineering, and production planning activities. Product design aligned with the design-to-cost concept can determine up to 70% of final costs; therefore, decisions made at the design stage are crucial for subsequent economic outcomes [8]. Moreover, effective management of organisational complexity requires a holistic approach by project teams and the avoidance of excessive specialisation, which often leads to fragmented decision-making processes and reduced managerial efficiency [5,8,23,32].

The results presented in this study are derived from a single industrial case and should therefore be interpreted as illustrative rather than statistically generalisable. The proposed method is primarily applicable to manufacturing enterprises operating under single-piece and small-batch production conditions, where product variety is high and material demand is highly diversified in terms of grades, dimensions, and forms.

From an organisational perspective, the method is particularly suitable for small and medium-sized enterprises (SMEs) and medium-sized industrial companies, in which material-management costs, inventory levels, and supplier relationships constitute a substantial share of total production costs. In very large-scale or highly standardised mass-production environments, where material variety is limited and economies of scale dominate cost structures, the potential benefits of the proposed approach may be reduced.

Regarding product mix, the method is best suited to products with a high share of material and machining costs, especially components, where alternative material substitution is technically feasible without violating quality or functional requirements.

## 5. Conclusions

The developed method for assessing the manufacturability of design decisions in the context of reducing material diversity enables:

- The identification of feasible applications of alternative materials;
- The estimation of their impact on material costs, processing costs, and production organisation;
- A significant reduction in material assortment;
- A decrease in material-management costs and overall production costs.

The conducted research confirms that limiting the number of material grades and standard dimensions can be one of the key tools for improving economic efficiency in machinery manufacturing enterprises, particularly under single-piece and small-batch production conditions. Such actions, however, must be preceded by a detailed analysis of the impact of the proposed changes on the entire manufacturing system, using comprehensive methods for modelling costs and order lead times [4,5,16,20,32,33,34,35,36].

Future work will focus on extending the model by incorporating additional decision criteria (e.g., energy and environmental indicators), strengthening substitute validation through explicit material-property constraints, and improving time estimation by calibrating machining coefficients using shop-floor data. Furthermore, supplier lead times and material price volatility will be integrated into a robust multi-criteria optimisation framework, and the approach will be validated across additional enterprises and families of semi-finished products.

## Figures and Tables

**Table 2:** Excerpt from the preliminary material requirements plan. ‘Date of demand’—planned requirement date derived from the production schedule; ‘Index’—ERP material code; ‘Name’—material grade and form (semi-finished product specification); ‘Gross weight’—total required mass [kg] for the planning period, calculated from BOM quantities and material standards.

Date of Demand	Index	Name	Gross Weight [kg]
26 March	0455001130000018	Round bar 1H18N9T, D = 140 mm, rolled	14.52
27 March	0455001200000005	Round bar 3H13, D = 110 mm, rolled	4.48
27 March	0455001270000012	Round bar 55, D = 55 mm, rolled	0.56
25 March	0455001270000004	Round bar 55 D = 50 mm, rolled	0.31
27 March	0455001270000010	Round bar 55 D = 35 mm, rolled	0.17
25 March	0455001000000009	Round bar 18HGT D = 30 mm, rolled	0.88
…	…	…	…

**Table 3:** Excerpt from the binary alternative materials matrix: 1—substitution feasible/allowed; 0—substitution not allowed.

Semi-Finished Products: Substitutes	Round Bar 18HGT D = 30 mm, Rolled	Round Bar 1H18N9T D = 140 mm, Rolled	Round Bar 3H13 D = 110 mm, Rolled	Round Bar 55 D = 35 mm, Rolled	Round Bar 55 D = 50 mm, Rolled	Round Bar 55 D = 55 mm, Rolled
Round bar 18HGT D = 30 mm, rolled	1	0	0	0	0	0
Round bar 1H18N9T D = 140 mm, rolled	0	1	1	0	0	0
Round bar 3H13 D = 110 mm, rolled	0	0	1	0	0	0
Round bar 55 D = 35 mm, rolled	0	0	0	1	0	0
Round bar 55 D = 50 mm, rolled	0	0	0	1	1	0
Round bar 55 D = 55 mm, rolled	0	0	0	1	1	1

**Table 4:** Corrected variants of alternative material application (excerpt).

Variant No.	Semi-Finished Products	Substitutes	Variant No.	Semi-Finished Products	Substitutes
0	0455001130000018	0455001130000018	11	0455001130000018	0455001130000018
	0455001200000005	0455001200000005		0455001200000005	0455001200000018
	0455001270000012	0455001270000012		0455001270000012	0455001270000012
	0455001270000004	0455001270000004		0455001270000004	0455001270000004
	0455001270000010	0455001270000010		0455001270000010	0455001270000010
	0455001000000009	0455001000000009		0455001000000009	0455001000000009
1	0455001130000018	0455001130000018	29	0455001130000018	0455001130000018
	0455001200000005	0455001200000018		0455001200000005	0455001200000005
	0455001270000012	0455001270000012		0455001270000012	0455001270000012
	0455001270000004	0455001270000012		0455001270000004	0455001270000012
	0455001270000010	0455001270000012		0455001270000010	0455001270000012
	0455001000000009	0455001000000009		0455001000000009	0455001000000009

**Table 5:** Excerpt from the analysis of changes in direct material costs.

**Requirement for the Semi-Finished Product**	**Alternative Semi-** **Finished Product**	${MS}_{y}$ [kg/pcs]	$p_{y}$ [PLN/kg]	$C_{DMy}$ [PLN/pcs]	${MS}_{w}$ [kg/pcs]	$p_{w}$ [PLN/kg]	$C_{DMw}$ [PLN/pcs]	${\Delta C}_{DM}$ [PLN/pcs]
Round bar 1H18N9T D = 140 mm, rolled	Round bar 1H18N9T D = 140 mm, rolled	14.52	29.70	431.24	14.52	29.70	431.24	0.00
Round bar 3H13 D = 110 mm, rolled	Round bar 1H18N9T D = 140 mm, rolled	4.48	23.40	104.83	7.25	29.70	215.32	110.49
Round bar 55 D = 55 mm, rolled	Round bar 55 D = 55 mm, rolled	0.56	1.53	0.86	0.56	1.53	0.86	0.00
Round bar 55 D = 50 mm, rolled	Round bar 55 D = 55 mm, rolled	0.31	1.65	0.51	0.37	1.53	0.56	0.05
Round bar 55 D = 35 mm, rolled	Round bar 55 D = 55 mm, rolled	0.17	6.45	1.10	0.42	1.53	0.64	–0.46
Round bar 18HGT D = 30 mm, rolled	Round bar 18HGT D = 30 mm, rolled	0.88	11.46	10.08	0.88	11.46	10.08	0.00

**Table 6:** Excerpt from the analysis of changes in processing activity costs.

**Part**	**Operation**	Seq.No.	${ST}_{My}$ [h/pcs]	${ST}_{Mw}$ [h/pcs]	Cmh [PLN/h]	$C_{APy}$ [PLN/pcs]	$C_{APw}$ [PLN/pcs]	${\Delta C}_{P}$ [PLN/pcs]
6531702	SCP cutting	10	0.54	0.75	69.53	37.55	52.15	14.60
6531702	STT turning	20	1.26	1.52	65.57	82.62	99.67	17.05

**Table 7:** Additional operation costs and total processing costs—excerpt.

Requirement for the Semi-Finished Product	Alternative Semi- Finished Product	$C_{APad.}$ [PLN/pcs]	${\Delta C}_{P}$ [PLN/pcs]	$C_{P}$ [PLN/pcs]
Round bar 1H18N9T D = 140 mm, rolled	Round bar 1H18N9T D = 140 mm, rolled	0.00	0.00	0.00
Round bar 3H13 D = 110 mm, rolled	Round bar 1H18N9T D = 140 mm, rolled	136.35	31.65	168.00
Round bar 55 D = 55 mm, rolled	Round bar 55 D = 55 mm, rolled	0.00	0.00	0.00
Round bar 55 D = 50 mm, rolled	Round bar 55 D = 55 mm, rolled	43.29	1.05	44.34
Round bar 55 D = 35 mm, rolled	Round bar 55 D = 55 mm, rolled	43.29	4.47	47.76
Round bar 18HGT D = 30 mm, rolled	Round bar 18HGT D = 30 mm, rolled	0.00	0.00	0.00

**Table 8:** Material management costs—excerpt.

	**Requirement for the Semi-Finished Product**	**Alternative Semi-** **Finished Product**	${\Delta C}_{DM}$ [PLN/pcs]	$C_{P}$ [PLN/pcs]	${\Delta C}_{AMM}$ [PLN/pcs]	${\Delta C}_{T}$ [PLN/pcs]
**+**	Round bar 1H18N9T D = 140 mm, rolled	Round bar 1H18N9T D = 140 mm, rolled	0.00	0.00	0.00	0.00
**−**	Round bar 3H13 D = 110 mm, rolled	Round bar 1H18N9T D = 140 mm, rolled	110.49	168.00	−695.67	−417.18
	**Activity**	**Cost driver**	$C_{AMMtotal}$ **[PLN]**	**Measure**	$C_{AMMunit}$**[PLN/pcs**]	
	Steel order	Number of items on GR	8033.34	153.50	52.33	
	Steel transport	Number of items on GR	54,360.75	153.50	354.14	
	Steel receipt	Number of items on GR	27,937.59	153.50	182.00	
	Steel storage	Number of warehouse items	23,370.36	218	107.20	
**+**	Round bar 55 D = 55 mm, rolled	Round bar 55 D = 55 mm, rolled	0.00	0.00	0.00	0.00
**+**	Round bar 55 D = 50 mm, rolled	Round bar 55 D = 55 mm, rolled	0.05	44.34	−267.70	−223.31
**+**	Round bar 55 D = 35 mm, rolled	Round bar 55 D = 55 mm, rolled	−0.46	47.76	−611.61	−564.31
**+**	Round bar 18HGT D = 30 mm, rolled	Round bar 18HGT D = 30 mm, rolled	0.00	0.00	0.00	0.00

**Table 9:** Selected solution—excerpt.

Index	Name	Gross Weight [kg]
0455001130000018	Round bar 1H18N9T D = 140 mm, rolled	20.22
0455001270000012	Round bar 55 D = 55 mm, rolled	1.16
0455001000000009	Round bar 18HGT D = 30 mm, rolled	0.88
…	…	…

## Data Availability

The original contributions presented in this study are included in the article. Further inquiries can be directed to the corresponding author.

## References

[1] Grznár P., Burganová N., Mozol Š., Mozolová L. (2023). A Comprehensive Digital Model Approach for Adaptive Manufacturing Systems. Appl. Sci..

[2] Haas K., Schuck H., Mücke T., Ovtcharova J. (2016). A holistic product lifecycle management approach to support design by machine data. Procedia CIRP.

[3] Kuric I. New methods and trends in product development and planning. Proceedings of the 1st International Conference on Quality and Innovation in Engineering and Management.

[4] Maskell B.H., Baggaley B., Grasso L. (2011). Practical Lean Accounting: A Proven System for Measuring and Managing the Lean Enterprise.

[5] Nicholas J. (2018). Lean Production for Competitive Advantage: A Comprehensive Guide to Lean Methods and Management Practices.

[6] Project Management Institute (2021). A Guide to the Project Management Body of Knowledge (PMBOK^®^ Guide) and the Standard for Project Management.

[7] Vavrík V., Fusko M., Bučková M., Gašo M., Furmannová B., Štaffenová K. (2022). Designing of Machine Backups in Reconfigurable Manufacturing Systems. Appl. Sci..

[8] Anderson D.M. (2020). Design for Manufacturability: How to Use Concurrent Engineering to Rapidly Develop Low-Cost, High-Quality Products for Lean Production.

[9] Chwastyk P., Kołosowski M. (2014). Estimating the cost of the new product in development process. Procedia Eng..

[10] Harris C., Harris R., Streeter C. (2017). Lean Supplier Development: Establishing Partnerships and True Costs throughout the Supply Chain.

[11] Matuszek J., Kaczmar-Kolny E., Byrdy Ł. (2023). The Method of Determining the Technical Costs of Manufacturing Products. Found. Manag..

[12] Aguilar-Virgen Q., Castañeda-González M., Marquez-Benavides L., Gonzalez-Vazquez J., Taboada-González P. (2021). Concurrent Engineering Model for the Implementation of New Products in the Textile Industry: A Case Study. Appl. Sci..

[13] Modrák V. (2023). Assessment of Product Variety Complexity. Entropy.

[14] Pakkanen J., Huhtala P., Juuti T., Lehtonen T. (2016). Achieving benefits with design reuse in manufacturing industry. Procedia CIRP.

[15] Steimer C., Aurich J.C. (2016). Analysis of information interdependencies between product development and manufacturing system planning in early design phases. Procedia CIRP.

[16] Mascitelli R. (2007). The Lean Product Development Guidebook: Everything Your Design Team Needs to Improve Efficiency and Slash Time-to-Market.

[17] Więcek D., Więcek D., Kuric I. (2019). Cost estimation methods of machine elements at the design stage in unit and small lot production conditions. Manag. Syst. Prod. Eng..

[18] Seal W.B., Rohde C., Garrison R.H., Noreen E.W. (2019). Management Accounting.

[19] Saniuk A., Saniuk S., Witkowski K. Using activity based costing in the metalworking processes. Proceedings of the 19th International Metallurgical and Materials Conference METAL 2011.

[20] Quesado P., Silva R.J.C. (2021). Activity-Based Costing (ABC) and Its Implication for Open Innovation. J. Open Innov. Technol. Mark. Complex..

[21] Mohd Zaini S.N.A., Abu M.Y. (2023). Implementing Time-Driven Activity-Based Costing for Unused Capacity Measurement in Local University. Sustainability.

[22] Więcek D., Plinta D., Radwan K., Burduk A., Batako A.D.L., Machado J., Wyczółkowski R., Dostatni E., Rojek I. (2024). The Role of Throughput Accounting in Making Decision in Small Batch Production Environment. Intelligent Systems in Production Engineering and Maintenance III. ISPEM 2023.

[23] Więcek D., Więcek D. (2017). The influence of the methods of determining cost drivers values on the accuracy of costs estimation of the designed machine elements. Proceedings of the International Conference on Information Systems Architecture and Technology.

[24] Micieta B., Staszewska J., Kovalsky M., Krajcovic M., Binasova V., Papanek L., Antoniuk I. (2021). Innovative System for Scheduling Production Using a Combination of Parametric Simulation Models. Sustainability.

[25] Więcek D., Burduk A., Kuric I. (2019). The use of ANN in improving efficiency and ensuring the stability of the copper ore mining process. Acta Montan. Slovaca.

[26] Płonka S., Ogiński L. (2014). Multicriterial optimisation of the manufacturing process of a spindle working in a ring spinning frame. Fibres Text. East. Eur..

[27] Grznár P., Gregor M., Mozol Š., Krajčovič M., Dulina L., Gašo M., Major M. (2019). A System to Determine the Optimal Work-in-Progress Inventory Stored in Interoperation Manufacturing Buffers. Sustainability.

[28] Oztürk D. (2017). Technological transformation of manufacturing by smart factory vision: Industry 4.0. Int. J. Dev. Res..

[29] Dohn K., Gumiński A. (2012). The identification of knowledge management tools in the context of the range of functionalities of computer system. Inf. Syst. Manag..

[30] Pride W.M., Hughes R.J., Kapoor J.R. (2014). Foundations of Business.

[31] Relich M., Nielsen I., Gola A. (2022). Reducing the Total Product Cost at the Product Design Stage. Appl. Sci..

[32] Juniani A.I., Singgih M.L., Karningsih P.D. (2022). Design for Manufacturing, Assembly, and Reliability: An Integrated Framework for Product Redesign and Innovation. Designs.

[33] Gregor T., Krajčovič M., Więcek D. (2017). Smart connected Logistics. Procedia Eng..

[34] Krajčovič M., Furmannová B., Grznár P., Furmann R., Plinta D., Svitek R., Antoniuk I. (2021). System of Parametric Modelling and Assessing the Production Staff Utilisation as a Basis for Aggregate Production Planning. Appl. Sci..

[35] Krajčovič M., Bastiuchenko V., Furmannová B., Botka M., Komačka D. (2024). New Approach to the Analysis of Manufacturing Processes with the Support of Data Science. Processes.

[36] McNerney J., Farmer J.D., Redner S., Trancik J.E. (2011). Role of design complexity in technology improvement. Proc. Natl. Acad. Sci. USA.

